# Time to Treatment and In-Hospital Major Adverse Cardiac Events Among Patients With ST-Segment Elevation Myocardial Infarction Who Underwent Primary Percutaneous Coronary Intervention (PCI) According to the 24/7 Primary PCI Service Registry in Iran: Protocol for a Cross-Sectional Study

**DOI:** 10.2196/13161

**Published:** 2019-03-01

**Authors:** Younes Nozari, Babak Geraiely, Kian Alipasandi, Arash Jalali, Negar Omidi, Hassan Aghajani, Alimohammad Hajizeinali, Mohammad Alidoosti, Hamidreza Pourhoseini, Mojtaba Salarifar, Alireza Amirzadegan, Ebrahim Nematipour, Mahin Nomali

**Affiliations:** 1 Department of Interventional Cardiology Tehran Heart Center Tehran University of Medical Sciences Tehran Islamic Republic of Iran; 2 Department of Cardiology School of Medicine, Tehran Heart Center Tehran University of Medical Sciences Tehran Islamic Republic of Iran; 3 Department of Research and Biostatistics Tehran Heart Center Tehran University of Medical Sciences Tehran Islamic Republic of Iran; 4 Department of Cardiology Tehran Heart Center Tehran University of Medical Sciences Tehran Islamic Republic of Iran; 5 Endocrinology and Metabolism Research Center Endocrinology and Metabolism Clinical Sciences Institute Tehran University of Medical Sciences Tehran Islamic Republic of Iran

**Keywords:** time to treatment, ST elevation myocardial infarction, percutaneous coronary intervention, cross-sectional studies, registries, Iran

## Abstract

**Background:**

Patients with ST-segment elevation myocardial infarction (STEMI) experience major adverse cardiac events (MACEs) following primary percutaneous coronary intervention (PCI). Although the relationship between time to treatment (eg, door-to-balloon time, symptom onset-to-balloon time, and symptom onset-to-door time) and 1-month all-cause mortality was assessed previously, its relationship with in-hospital MACEs and the effect of some clinical characteristics on this relationship were not considered. Furthermore, previous studies that were conducted in developed countries with a different quality of care cannot be applied in Iran, as Iran is a developing country and the studies were not performed according to the 24/7 primary PCI service registry.

**Objective:**

The objective of this study protocol is to determine the relationship between time to treatment and in-hospital MACEs.

**Methods:**

This cross-sectional study will take place at the Tehran Heart Center (THC), which is affiliated with Tehran University of Medical Sciences (TUMS) in Tehran, Iran. Data related to patients with STEMI, who underwent primary PCI between March 2015 and March 2019, that have been prospectively recorded in the THC’s 24/7 primary PCI service registry will be analyzed. The study outcome is the occurrence of in-hospital MACEs. Data analysis will be conducted using SPSS for Windows, version 16.0 (SPSS Inc). We will perform chi-square tests, independent-samples t tests, or the Mann-Whitney U test, as well as univariate and multivariate binary logistic regression with a significance level of less than .05 and 95% CI for odds ratios.

**Results:**

From March 2015 to September 2017, 1586 patients were included in the THC service registry, consecutively. We will conduct a retrospective analysis of this registry on patient entries between March 2015 and March 2019 and data will be analyzed and published by the end of 2019.

**Conclusions:**

To our knowledge, this is the first observational study based on the 24/7 primary PCI service registry in Iran. The findings of this study may reveal current problems regarding time to treatment in STEMI management in the THC. Results from this study may help determine appropriate preventive strategies that need to be applied in order to reduce time-to-treatment delays and improve patients’ outcomes following primary PCI in the setting of STEMI at the THC and similar clinical centers.

**International Registered Report Identifier (IRRID):**

DERR1-10.2196/13161

## Introduction

Cardiovascular diseases continue to be a main cause of premature mortality and long-term disability worldwide [1]. In addition, ischemic heart disease was the first leading cause of death globally among men and women in 2013 [2]. In 2015, acute myocardial infarction was estimated at 7.92 million cases globally [1]. In Iran, one of the largest Middle Eastern countries in South-Western Asia, there is a high and increasing prevalence of acute myocardial infarction [3], with a variation of 5% to 15% in different cities [4-6]. According to a review study in 2016, the in-hospital case-fatality rate of myocardial infarction was 12.1%; ST-segment elevation myocardial infarction (STEMI) and being over 84 years of age were contributing factors [7].

Primary percutaneous coronary intervention (PCI) is the preferred reperfusion strategy for patients with STEMI [8] because of the lower rate of total stroke, hemorrhagic stroke, and reinfarction; the increase in patency of infarct-related artery; and the improvement in the in-hospital and long-term survival rate [9]. In addition, it can be a reliable substitute for patients with thrombolytic therapy contraindication [10]. Furthermore, it can be performed faster and result in a lower mortality rate if performed in high-volume hospitals [11]. However, patients with STEMI undergoing primary PCI may experience major adverse cardiac events (MACEs) [12].

Although the recent guidelines have emphasized performing reperfusion strategy for patients with STEMI in a timely manner [13], longer total times have been found [14]. Time to treatment includes symptom-to-door time (ie, time between symptom onset to hospital arrival), door-to-balloon time (ie, time between hospital arrival and balloon inflation), and symptom-to-balloon time (ie, time between symptom onset and balloon inflation) [15]. Longer time to treatment may have an impact on outcome following primary PCI [16]. Thus, time-to-treatment delays should be acknowledged as a key issue [8] and as the easiest audit index of care quality in STEMI management; it should be recorded and reviewed regularly in every health system providing care to patients with STEMI [17].

Door-to-balloon time of less than 90 minutes has been considered as the maximum target time for primary PCI [17]. Previous studies found that door-to-balloon time was associated with better in-hospital outcomes and long-term survival [18-23]. In the Korea Acute Myocardial Infarction Registry (KAMIR), the rate of achieving the target door-to-balloon time increased from 70.3% in 2008 to 90.2% in 2011 [15]. In Iran, according to the Iranian Project for Assessment of Coronary Events 2 (IPACE2) study, although Iranian patients with STEMI received in-hospital reperfusion in a timely fashion, there were long patient delays [24]. However, the effects of these delays on MACEs among patients with STEMI undergoing primary PCI in health care settings of a developing country such as Iran is still unknown.

In contrast, despite improvements in door-to-balloon time over the years, several studies demonstrated no improvement in clinical outcomes and survival rates of patients who underwent primary PCI [15,25-28]. These studies often focused on door-to-balloon time, while symptom-to-balloon time and total ischemic time, as better predictors of clinical outcomes, had not been considered [29].

Although previous studies indicated no relationship between door-to-balloon time and 1-year MACEs [21,26], there is a controversial relationship between symptom-to-balloon time and in-hospital and 1-year MACEs in the literature. Despite the strong relationship between symptom-to-balloon time and 1-year MACEs, no relationship was found between symptom-to-balloon time and in-hospital MACEs [29,30].

Because time to treatment can be important for predicting clinical outcomes, the relationship between time to treatment and 1-month all-cause mortality was assessed by Kim et al in 2017 for the first time [15]. However, its relationship with in-hospital MACEs and the effect of some clinical characteristics on this relationship were not considered. Furthermore, previous studies that were conducted in developed countries with a different quality of care cannot be applied in Iran, as Iran is a developing country and the studies were not performed according to the 24/7 primary PCI service registry.

There is no information on the relationship between time to treatment and in-hospital MACEs among patients with STEMI undergoing primary PCI in Iran. Such information is necessary for health care systems to identify sources of time delays, to help them plan better, and to allow them to apply preventive strategies for improving clinical outcomes following primary PCI in STEMI management. Therefore, this study has been designed to determine the relationship between time to treatment and in-hospital MACEs among patients with STEMI who have undergone primary PCI according to the Tehran Heart Center’s (THC) 24/7 primary PCI service registry in Iran.

## Methods

### Study Design

This is a cross-sectional study that has been approved by the Institutional Review Board (#9511171022) and the Research Ethics Committee (REC) of Tehran University of Medical Sciences (TUMS), Tehran, Iran, on July 25, 2018 (approval ID: IR.TUMS.MEDICINE.REC.1397.290). This study protocol is based on the Strengthening the Reporting of Observational Studies in Epidemiology (STROBE) statement [31].

### Setting

The study will take place at the THC affiliated with TUMS. The THC is a “cardiac center of excellence” and provides medical services by full-time specialists and well-trained nursing staff; it is considered one of the best-equipped diagnostic and therapeutic cardiology centers in Iran and in the region. From 2001 to the end of 2017, about 1,300,000 outpatient visits, 280,000 hospitalizations, and 35,000 angioplasties have been recorded for the THC [32]. In addition, primary PCI has been performed at the THC since 2004, and 2380 patients underwent primary PCI as of September 2017 (Third Report of the Databank of the THC, 2017 [internal report]).

### Participants

Patients of both genders, over 18 years of age, experienced STEMI and received primary PCI within the recommended time interval (ie, 120 minutes from STEMI diagnosis) [13] through the standard technique and without bolus administration of fibrinolysis in one of six catheterization laboratories in the THC; those with complete registered data in the THC’s 24/7 primary PCI service registry from March 2015 to March 2019 will be included in the study. Thus, patients with incomplete time-to-treatment data, such as symptom onset, hospital arrival, and balloon time, will be excluded from the study.

### Variables

Variables such as demographic and clinical characteristics and study outcome will be used for statistical analysis (see Table 1). Study outcome will be the occurrence of in-hospital MACEs, which will be measured during hospital admission following primary PCI. The occurrence of in-hospital MACEs is a composite index of different elements (eg, myocardial infarction, stroke, cardiac death, target vessel revascularization, and target lesion revascularization), which have been defined in Textbox 1.

### Data Sources and Measurement

We will use the THC 24/7 primary PCI service registry as the main data source in our study. The THC joined this service registry in March 2015. At first, it was conducted at the THC as a pilot scheme for several months. Afterward, in September 2015, it was applied as a default treatment strategy for patients with acute STEMI (Third Report of the Databank of the THC, 2017 [internal report]).

The THC 24/7 primary PCI service registry was approved by the REC affiliated with TUMS and informed consent was obtained from all patients for data collection and follow-ups. Data related to patients with STEMI who underwent primary PCI in one of the six catheterization laboratories in the THC were collected prospectively through a STEMI management registry form. Data were collected by trained emergency department nursing staff at admission time, catheterization laboratory nursing staff, and interventional cardiology fellows; data will be entered into the service registry by research staff on a weekly basis. Patients who expired before beginning the procedure were not included in the registry.

The STEMI management registry form, which is completed prospectively, includes data related to patient demographics (eg, name, birth date, identification code, and gender); admission (eg, mode of patient’s transfer, symptom onset time, first medical contact, first electrocardiogram [ECG] in ambulance, and door time); electrocardiographic assessment (eg, infarcted territories, first hospital ECG time, STEMI ECG time, and STEMI ECG verification time); initial reperfusion therapy (eg, transfer to catheterization laboratory, only fibrinolysis, or no reperfusion with reason); procedure in the catheterization laboratory (eg, arrival time, device time, type of intervention performed, infarct-related artery, initial and final Thrombolysis in Myocardial Infarction [TIMI] flow, and stent thrombosis); and additional treatments from symptom onset to coronary intervention (eg, transvenous or external pacemaker, ventilator support, inotropes, intra-aortic balloon pump, left ventricular assistive device, Impella, cardioversion and defibrillator, and cardiopulmonary resuscitation). In this registry, 30-day, 6-month, and 1-year follow-ups for outcome assessment will be conducted by dedicated research staff via phone calls and outpatients’ clinical record review.

### Bias

As the 24/7 primary PCI service registry has been conducted in the THC since March 2015, there may be a considerable number of patients with missing treatment times. Excluding these patients from the analysis may introduce selection bias. As an additional analysis, we will compare baseline characteristics between excluded and included groups in order to find any discrepancy and evidence of selection bias. Moreover, to minimize the bias, strict control of quality and professional supervision on the service registry will be applied.

### Study Size

We will conduct a retrospective analysis on patient entries in this service registry between March 2015 and March 2019. From March 2015 to September 2017, data related to 1586 patients were recorded in the THC’s 24/7 primary PCI service registry (Third Report of the Databank of the THC, 2017 [internal report]); we expect to reach 2500 patients by March 2019.

**Table 1:** Demographic and clinical variables and outcome included in the study.

Variables		Description	Presentation
**Demographic characteristics**			
	Age	Age in years at time of admission	Mean (SD); <65 years or ≥65 years, n (%)
	Sex	Male or female gender	Male or female, n (%)
	Current smoker	Smoking status at time of admission	Yes or no, n (%)
**Clinical characteristics**			
	Past medical history	Previous myocardial infarction, PCI^a^, and coronary artery bypass graft surgery	Yes or no, n (%)
	Comorbidities	Having diabetes mellitus, hypertension, and hyperlipidemia at time of admission based on patient or family self-report	Yes or no, n (%)
	Family history of cardiovascular diseases	Having family history of cardiovascular diseases based on patient or family self-report	Yes or no, n (%)
	Emergency medical service user	Transferred to the THC^b^ by emergency medical service	Yes or no, n (%)
	First medical contact	Emergency medical service arrival time at scene or patient arrival time at emergency department of the THC	Median (IQR^c^), minutes
	First ECG^d^ time	Time that first ECG was taken at the THC	Median (IQR), minutes
	STEMI^e^ ECG time	Time that STEMI was detected on ECG by emergency department staff	Median (IQR), minutes
	STEMI verification time	Time STEMI was verified by the emergency department physician	Median (IQR), minutes
	24/7 code time	Time that the 24/7 code was activated at the THC	Median (IQR), minutes
	Number of diseased vessels	Number of single, double, and triple diseased vessels	Single, double, and triple diseased vessels, n (%)
	Infarct-related artery	Infarct-related artery	Left coronary artery, left anterior descending artery, left circumflex artery, right coronary artery, or posterior descending artery, n (%)
	Infarcted territory	Territory of myocardial infarction according to the ECG	Anterior, posterior, inferior, or lateral, n (%)
	Killip class	Patients’ risk classification for development of heart failure in order to predict mortality	I, II, III, or IV, n (%)
	Preprimary PCI TIMI^f^ flow	Level of coronary blood flow before primary PCI	0, 1, 2, or 3, n (%)
	Postprimary PCI TIMI flow	Level of coronary blood flow following primary PCI	0, 1, 2, or 3, n (%)
	Symptom-to-door time	Time from self-reported onset of symptoms to time of hospital arrival	Median (IQR), minutes; <90 minutes, n (%)
	Door-to-balloon time	Time from hospital arrival to time of reperfusion (ie, wire crossing)	Median (IQR), minutes; <90 minutes, n (%)
	Symptom-to-balloon time	Time from self-reported onset of symptoms to time of reperfusion (ie, wire crossing)	Median (IQR), minutes; <180 minutes, n (%)
	Procedural supports	Devices and medications used before, during, and after primary PCI	Pacemaker, mechanical ventilation, intra-aortic balloon pump, inotropes, cardioversion, and defibrillator, n (%)
	Stent type	Type of stent used during reperfusion	Bare metal stent, drug-eluting stent, or no stent, n (%)
**Outcome**			
	In-hospital MACEs^g^	Composite of myocardial infarctions, stroke, and cardiac death following primary PCI before hospital discharge	Occurrence of MACEs: yes or no; total MACEs, n (%)

^a^PCI: percutaneous coronary intervention.

^b^THC: Tehran Heart Center.

^c^IQR: interquartile range.

^d^ECG: electrocardiogram.

^e^STEMI: ST-segment elevation myocardial infarction.

^f^TIMI: Thrombolysis in Myocardial Infarction.

^g^MACE: major adverse cardiovascular event.

**The composition of different elements included in the study.**

- Myocardial infarction: clinical evidence of acute myocardial ischemia and detection of a rise and/or fall of cardiac troponin values with at least one value above the 99th percentile upper limit and at least one of the following:
  - Symptoms of myocardial ischemia
  - New ischemic electrocardiogram changes
  - Development of pathological Q waves [33]
- Stroke: a new focal neurological deficit taking longer than 24 hours and confirmed by imaging [29]
- Target vessel revascularization: either percutaneous coronary intervention or coronary artery bypass graft surgery of the target vessel as the main coronary vessel proximal and distal to the target lesion [34]
- Target lesion revascularization: either percutaneous coronary intervention or coronary artery bypass graft surgery of the target vessel due to restenosis and other complications related to target lesion, which includes the treated segment from 5 mm proximal to the stent to 5 mm distal to the stent [34]
- Cardiac death: any death related to myocardial infarction, cardiac arrhythmia, and heart failure [26]

### Statistical Methods

We will conduct statistical analysis using the statistical package SPSS for Windows, version 16.0 (SPSS Inc). Categorical variables will be reported as numbers and percentages and will be compared by chi-square test. In order to compare continuous variables, independent-samples *t* tests or Mann-Whitney U tests will be used and they will be reported as mean (SD) or median (interquartile range [IQR]). Univariate binary logistic regression analysis will be used to determine the relationship between time to treatment and in-hospital MACEs. Multivariate analysis will be performed to identify predictors of in-hospital MACEs by the binary logistic regression model. Each correlation between the variables will be expressed as an odds ratio with a 95% CI. All statistical tests will be set as two-tailed tests, with a significance level of less than .05.

## Results

Data from March 2015 to September 2017 related to 1586 patients with STEMI who underwent primary PCI in the THC have been recorded consecutively in the THC service registry. Data analysis of this service registry will be conducted retrospectively on patient entries between March 2015 to March 2019. Data will be analyzed and published by the end of 2019.

## Discussion

As the first study on the 24/7 primary PCI service registry in Iran, it may reveal current problems regarding time to treatment in STEMI management at the THC. To our knowledge, this is the first observational study of the relationship between time to treatment and in-hospital MACEs according to the service registry in Iran. Therefore, the findings of this study may help us detect sources of delays—patient delays, system delays, or both. These findings may assist us in applying appropriate preventive strategies in order to reduce time-to-treatment delays and improve patients’ outcomes following primary PCI in the STEMI setting in the THC and similar clinical centers.

There are some limitations in this study. First, this is a single-center observational study. Thus, no causal relationship between time to treatment and in-hospital MACEs can be proven conclusively. Second, the study population consists of patients with STEMI who were treated with primary PCI. Therefore, the findings cannot be generalized to patients with STEMI who received thrombolytic therapy.

## Abbreviations

ECG: electrocardiogram
IPACE2: Iranian Project for Assessment of Coronary Events 2
IQR: interquartile range
KAMIR: Korea Acute Myocardial Infarction Registry
MACE: major adverse cardiac event
PCI: percutaneous coronary intervention
REC: Research Ethics Committee
STEMI: ST-segment elevation myocardial infarction
STROBE: Strengthening the Reporting of Observational Studies in Epidemiology
THC: Tehran Heart Center
TIMI: Thrombolysis In Myocardial Infarction
TUMS: Tehran University of Medical Sciences
